# Fever following an Epidural Blood Patch in a Child

**DOI:** 10.1155/2012/753875

**Published:** 2012-09-16

**Authors:** Agnes I. Hunyady, Corrie T. M. Anderson, John D. Kuratani, Anjana Kundu

**Affiliations:** ^1^Department of Anesthesiology and Pain Medicine, Seattle Children's Hospital, School of Medicine, University of Washington, Seattle, WA 98105-0371, USA; ^2^Seattle Children's Hospital, School of Medicine, University of Washington, Seattle, WA 98105-0371, USA

## Abstract

There is increasing evidence that children suffer from the consequences of spontaneous or iatrogenic intracranial hypotension. Pediatric epidural blood patch is gaining popularity because of its ability to alter cerebrospinal fluid dynamics and to alleviate headaches attributed to low cerebrospinal fluid pressure. There is, however, still not enough data to document the safety profile of an epidural blood patch. Here we describe a case of a fever in a child temporally related to the administration of an epidural blood patch. This case depicts the dilemmas in making the diagnosis and instituting treatment for complications of this procedure in the pediatric population.

## 1. Introduction

Intracranial hypotension caused by ongoing spontaneous or iatrogenic cerebrospinal fluid (CSF) leakage can result in, not only severe, incapacitating headaches, but also life-threatening complications [1–3]. The popularity of pediatric neuraxial anesthetic and analgesic techniques has increased in recent times, and recent reviews have shown that the incidence of postdural puncture headache (PDPH) in children is higher than was previously thought [4, 5]. In addition there has been an increasing awareness in the neurosurgical community of the availability of the epidural blood patch (EBP) as a therapeutic tool. Given these two factors, referral of children with headaches attributed to low CSF pressure to pediatric anesthesiologist is likely to increase. While the available small number of reported cases suggests that EBP is effective in children as well, insufficient data exist regarding the safety profile of pediatric EBPs [6, 7].

 Adding to the scant body of knowledge about epidural blood patches in children we are reporting a case of fever in a child, possibly related to a medication and temporally coincident to the performance of an EBP. 

## 2. Case Report

A fourteen-year-old girl presented to her primary care physician with fever, photophobia, nausea, vomiting, meningeal signs, and loss of consciousness nine months after a motor vehicle accident that had resulted in frontal sinus fractures. She was treated empirically with ceftriaxone for presumed bacterial meningitis (her family refused lumbar puncture), and with acyclovir for documented herpes simplex infection in her oral cavity. Because of continuing intermittent CSF leak, she was transferred to Children's Hospital and Regional Medical Center. Computer tomography (CT) scan of her head showed sinusitis, bilateral dehiscence of the posterior frontal sinuses at the level of the anterior cranial fossa, and herniation of the frontal lobes into the frontal sinus skull defects. She subsequently underwent surgical debridement of the sinuses, elevation of old frontal sinus fractures, and closure of the dura with concurrent lumbar drain placement.

**Figure 1:**
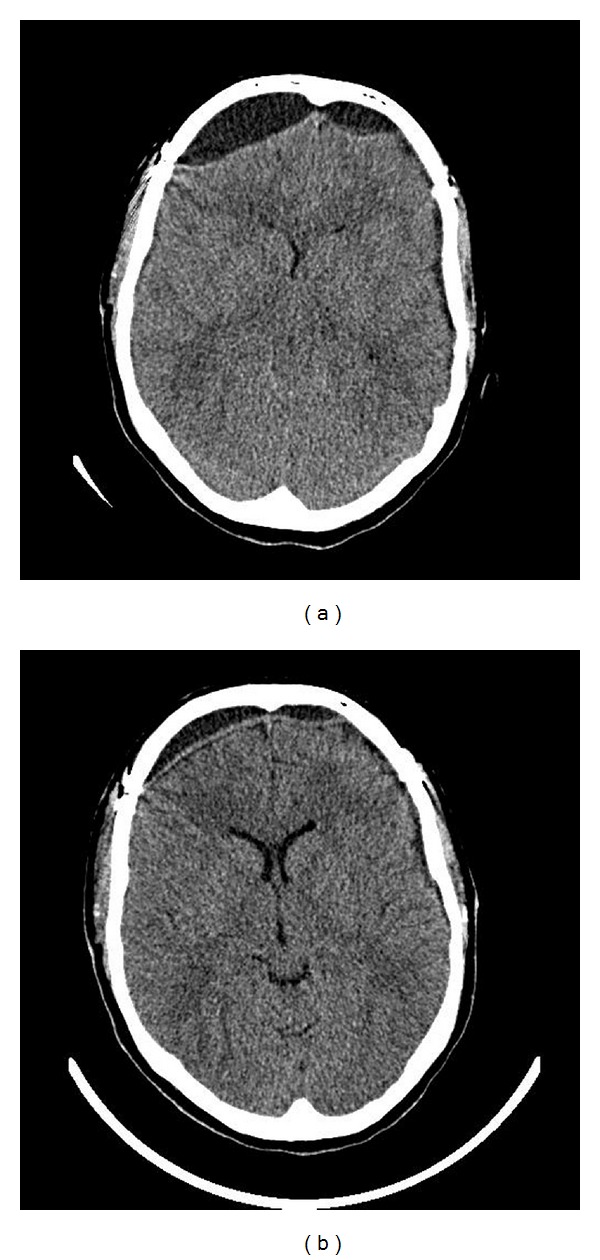
(a) Head CT of our patient the day before the EBP was performed. CSF leak resulted in large bifrontal fluid collections. (b) Head CT of the same patient the day after the EBP. The extradural CSF collection is significantly smaller in size. A possible explanation for this finding is the effect of the EBP on the intracranial pressure (as evidenced by the increased intraventricular space) prohibiting further leakage through an existent frontal tear on the dura.

A pruritic fine popular rash involving her arms and thighs, and gradually spreading to her face, complicated her perioperative course. The rash progressed into a confluent malar eruption that was thought to be related to ceftriaxone and prompted several changes in her antimicrobial regimen. Following her operation, her fevers and rash resolved, her headache improved, and her CT scan was reassuring. Yet, after removal of the lumbar drain, she developed severe postural headache accompanied by nausea, vomiting, and refusal to ambulate. Her CT scan showed bifrontal extradural CSF collection (Figure 1(a)). The pain service was requested to place an EBP. 

Our assessment revealed an otherwise healthy afebrile young teenage girl in moderate distress and a normal neurological exam. She had no signs of generalized or local infections, her coagulation studies were normal, and all her blood and CSF cultures since her initial presentation were negative. She was still receiving cefuroxime to finish her initial course of antibiotics.

After informed consent was obtained, the patient was taken to the operating room and anesthetized. Under sterile conditions an EBP was performed one interspace above the lumbar drain insertion site using 15 mL (0.3 mL/kg) blood. When the patient recovered from anesthesia, she complained of mild backache, but stated that her headache had resolved.

About 18 hours later, on a routine postprocedure visit the patient was noted to be flushed and febrile (maximum temperature 38.8°C). She had no headache at rest or with activity. She was able to ambulate for the first time in two weeks. The patient reported no nausea, vomiting, or meningeal signs. Her neurological exam and her complete blood count and differential were normal. Her head CT showed significant decrease of the size of the bifrontal extradural CSF collection (Figure 1(b)). After concerns were raised about the therapeutic intervention, discussion with the neurosurgical team followed, and expectant management with serial neurological checks was started. About three hours later, the patient reported pruritus on her arms and legs, and she was noted to have a fine maculopapular rash, very similar to the one she had shortly after admission. Review of her chart revealed that after the EBP her antibiotic was changed from cefuroxime to ceftriaxone by the resident on call to allow less frequent dosing. After discontinuation of the ceftriaxone and diphenhydramine administration, her rash improved and her fever resolved. Five days later she was discharged from the hospital and on followup two months later she continued to be healthy.

## 3. Discussion

Obstetrical patients with mild increases in temperature after the induction of epidural analgesia have been reported [8], however, pyrexia after an EBP is considered a sign of infection and a potentially life-threatening complication. An epidural abscess, meningitis, or other infectious causes have to be ruled out. 

Suspicion of meningitis warrants immediate diagnostic lumbar puncture, but a lumbar puncture (LP) can precipitate the exact same symptoms the EBP was meant to treat. Furthermore, it is contraindicated in the presence of an epidural abscess, which is one of the most feared complications of EBP. Emergency laminectomy and targeted antibiotic therapy are the key to decrease an otherwise high rate of mortality and morbidity. Diagnosis is usually made by magnetic resonance imaging, however, experience in detecting epidural abscesses in patients who have had an epidural blood patch is lacking. Also, detection of early infection of a patch might not be possible. Thus, LP through a potentially infected EBP carries the risk of transmitting pathogens from the extradural to the intradural space.

On rare occasions, other causes of pyrexia should be considered: coincidental septicemia following EBP has been described [9], so were other noninfectious causes, like drug- or chemically induced aseptic meningitis, mimicking bacterial meningitis [10, 11]. In this case, an obvious discrepancy between the clinical picture and the degree of pyrexia leads to deferral of invasive diagnostic procedures and consideration of other causes and resulted in a positive outcome with patient satisfaction. To our knowledge, this is the first reported case of drug fever temporally related to an EBP.

**Figure 2:**
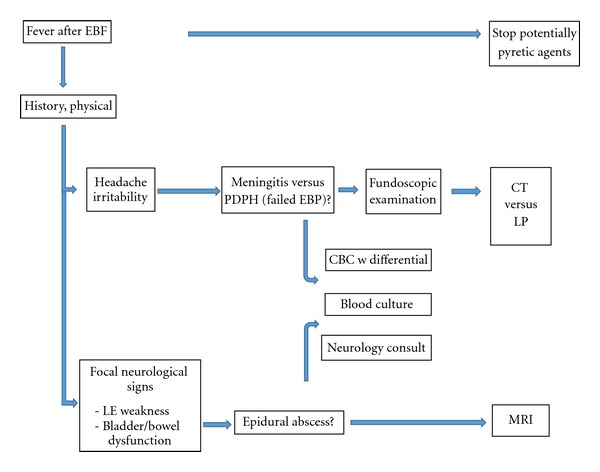
Stepwise diagnostic approach to fever after an EBP.

The recognition of an infectious complication of an EBP in a young child can be difficult. We propose a stepwise approach in the evaluation of a child with a fever following an epidural blood patch (Figure 2). A thorough history and physical examination is the first step, with careful attention to the neurologic exam. Focal signs involving the lower extremities or bowel/bladder incontinence would suggest a process affecting the lower spine, such as an epidural abscess. Headache and irritability are features of both PDPH and meningitis. A postural component to the headache would suggest low CSF pressure and PDPH from a failed EBP. The presence of papilledema on fundoscopic exam would indicate increased intracranial pressure and favor the diagnosis of meningitis. Because evaluation of the sensorium is not always straightforward in a young child, consultation with a neurologist is recommended. Secondly, any potentially pyretic agent or therapy should be halted. This would include heating blankets or warming covers. Next, the patient's white blood cell count with a differential cell count should be checked and blood cultures drawn. A magnetic resonance image of the spine to look for an epidural abscess is recommended especially if the fever occurs several days after the placement of the EBP or focal neurologic signs develop in the lower body. If fever and the suspicion of meningitis persist, a LP should be performed without delay. Surgical intervention will be necessary for an abscess.

In summary, EBP is a powerful therapeutic tool for the treatment of intracranial hypovolemia and consequent severe PDPH in children. We described a case of a fever in a child temporally coincident with an epidural blood patch, pointed out dilemmas in the diagnosis of infectious complications in the pediatric population, and proposed a stepwise diagnostic approach. The available literature on pediatric EBPs is scarce. Because of the negative behavioral response to medical interventions that the pediatric patient displays, pediatric EBPs are usually performed under anesthesia; we propose that data from the adult literature regarding safety and efficacy cannot be extrapolated.

Since EBP in children is infrequently performed, multiinstitutional data collection is necessary.
